# In absence of local adaptation, plasticity and spatially varying selection rule: a view from genomic reaction norms in a panmictic species (*Anguilla rostrata*)

**DOI:** 10.1186/1471-2164-15-403

**Published:** 2014-05-27

**Authors:** Caroline L Côté, Martin Castonguay, McWilliam Svetlana Kalujnaia, Gordon Cramb, Louis Bernatchez

**Affiliations:** 1 Institut de Biologie Intégrative et des Systèmes (IBIS), Université Laval, Québec G1V 0A6, Canada; 2 Institut Maurice-Lamontagne, Ministère des Pêches et des Océans, 850 Route de la Mer, Mont-Joli, Québec G5H 3ZH, Canada; 3 University of St Andrews, School of Biology, St Andrews, Fife KY16 9AJ, Scotland

**Keywords:** *Anguilla*, Panmixia, Trancriptome, Microarrays, Genomic reaction norm, Plasticity, Gene-environment interactions, Conservation

## Abstract

**Background:**

American eel (*Anguilla rostrata*) is one of the few species for which panmixia has been demonstrated at the scale of the entire species. As such, the development of long term local adaptation is impossible. However, both plasticity and spatially varying selection have been invoked in explaining how American eel may cope with an unusual broad scope of environmental conditions. Here, we address this question through transcriptomic analyses and genomic reaction norms of eels from two geographic origins reared in controlled environments.

**Results:**

The null hypothesis of no difference in gene expression between eels from the two origins was rejected. Many unique transcripts and two out of seven gene clusters showed significant difference in expression, both at time of capture and after three months of common rearing. Differences in expression were observed at numerous genes representing many functional groups when comparing eels from a same origin reared under different salinity conditions. Plastic response to different rearing conditions varied among gene clusters with three clusters showing significant origin-environment interactions translating into differential genomic norms of reaction. Most genes and functional categories showing differences between origins were previously shown to be differentially expressed in a study comparing transcription profiles between adult European eels acclimated to different salinities.

**Conclusions:**

These results emphasize that while plasticity in expression may be important, there is also a role for local genetic (and/or epigenetic) differences in explaining differences in gene expression between eels from different geographic origins. Such differences match those reported in genetically distinct populations in other fishes, both in terms of the proportion of genes that are differentially expressed and the diversity of biological functions involved. We thus propose that genetic differences between glass eels of different origins caused by spatially varying selection due to local environmental conditions translates into transcriptomic differences (including different genomic norms of reaction) which may in turn explain part of the phenotypic variance observed between different habitats colonized by eels.

## Background

Similar to other members of the freshwater eels of the genus *Anguilla*, the American eel (*Anguilla rostrata*, Lesueur 1817) represents one of the most scientifically intriguing cases of life history among fishes. Our recent findings unambiguously confirmed that this temperate euryhaline semelparous species comprises a single genetically panmictic population [1]. As for the European eel, American eel exhibits extreme inter-individual phenotypic variance among teleost fishes during their so-called yellow eel (juvenile) phase in terms of growth rate, length at maturity, morphology, sex ratio, fecundity, and other life history traits as well as pronounced regional variation in recruitment across the highly heterogeneous environments occupied by the postlarval up to adult stages [2-12].

It has been hypothesized that phenotypic variation observed across the species’ distribution range is associated with differential mortality caused by spatially varying selection associated to environmental heterogeneity [12,13]. Differential mortality is thus causing local genetic differences during a single generation and/or genetically-based intra-specific variation in migratory behavior and alternative habitat use [14]. However, this does not exclude a role for plasticity in coping with heterogeneous environments [15,16].

Understanding the relative importance of genetic versus plasticity factors represents a major challenge, and a first attempt to decipher both, we previously compared growth patterns in controlled conditions between young eels from two geographic origins [6]. This study revealed both an origin (genetic) and environmental (plastic) effect on growth, and suggested a possible origin-environment interaction. The relative roles of these factors on patterns of phenotypic variation in eels and the mechanistic nature of such variation can be investigated by means of gene expression analyses. Thus, although gene transcripts themselves represent discrete phenotypes, they may also result in externally visible (e.g. size, color) or more cryptic (e.g. physiological tolerance to environmental conditions, age at reproduction) phenotypes [7,17,18]. Phenotypic variance is ultimately controlled by gene expression, which is modulated by the interactions between the genotype and the environment. In absence of any local genetic differences between locations, and given panmixia, one expects that eels from different origins reared in similar environments should present similar gene expression profiles. By contrast, observing differences of transcription profiles between eels from different origins or different genomic norms of reaction across different rearing environments would suggest a possible role for genetic differences between eels from different origins, possibly resulting from spatially varying selection. Admittedly, epigenetic effects could also be involved in such a case. However, these can hardly be investigated in non-model species given the current state of the knowledge and tools at hands, although the role of epigenetics in controlling phenotypic variance in eel would obviously be of major interest in subsequent steps [19]. Finally, a better understanding of the underlying causes of phenotypic variation in the American eel across different environments is of outmost applied importance as this may impact on management decisions (e.g. translocations, defining management units) made towards improving its conservation [20,21].

The goal of this study was to perform a transcriptomic study by means of cDNA microarrays in order to assess the relative role of geographic origin, environment (salinity) and their interaction on transcription profiles observed both in natural and controlled conditions. Our results revealed that all three factors play a role in explaining variation of gene expression in American eel, thus indicating that phenotypic variation observed in the species is mainly but not purely plastic and therefore also involves genetically (and/or epigenetic) based differences between eels from different locations.

## Methods

This study was carried out in strict accordance with the recommendations in the Guide for the Care and Use of Laboratory Animals of the Canadian Council on Animal Care in science and all efforts were made to optimize environmental enrichment and avoid suffering. The protocol was approved by the Committee on the Good Care of Animal Experiments of Université Laval (Permit Number: 2007-165-1).

### Sampling and rearing conditions

We previously described the details of sampling and rearing conditions in Côté *et al*. [6]. In brief, glass eels were captured at two sampling locations in spring 2007: Mira river, 45°56’N 60°07’W, hereafter MR and Grande-Rivière-Blanche, 48°78’N 67°69’W, thereafter GRB. Glass eels correspond to the young life history stage at which they colonize coastal and continental waters following a 12 to 18 month oceanic migration from their spawning ground in the Sargasso Sea toward coastal or freshwater habitats [1].

Eels of both origins are characterized by contrasted life history in their natural settings. GRB represents eels from the St. Lawrence R. freshwater system which produces exclusively very large females [22] while MR represents a brackish environment were both males and small females occur in variable proportions and variable adult size (Dr. Martha Jones, Cape Breton University unpublished data). Glass eels of similar sizes were collected at the mouth of each river as soon as they approached the coast.

A sample of glass eels for each site was fixed for 24 h at 4°C in a saturated salt buffer immediately after collection in the field (T0) to preserve RNA and DNA integrity. The buffer was replaced within 24 h to a week and samples were stored at room temperature. The salt saturated buffer consisted of the following for one liter saturated salt solution; 40 mL of 0.5 M EDTA, 25 mL 1 M Sodium citrate, 700 g Ammonium sulfate and 935 mL ultrapure water, stirred on low heat combine until the Ammonium sulfate dissolves completely. Following cooling, pH was adjusted to 5.2 with H_2_SO_4_. Glass eel from both sites and captured at different time points were treated exactly in the same way, that is they were all randomly distributed into individual tanks after a same acclimation period of 12 days. This consisted in maintaining the freshwater condition at 3-5% and for the brackish water treatment, salinity was raised gradually from 3% to 22% with increment of approximately 2% per day. For each origin, glass eels were distributed and reared in 25 L aquaria at the Laboratoire de Recherche en Sciences Aquatiques (LARSA) at Université Laval for three months in two different salinity conditions: fresh (FW: 2–3 ppt) or brackish (BW: 20–22 ppt) water. At the end of this period, three samples per aquarium, for a total of 12 eels (hereafter named elvers) per origin and salinity conditions (total of 48 eels) were randomly collected. Individuals were sedated with 10% eugenol, measured for both total body length (LT ± 1 mm) and wet mass (W ± 0.1 mg), and frozen in liquid nitrogen.

### High quality RNA preparation

Extraction, conservation and retro-transcription of mRNA were processed as follows. Each sample, consisting of whole body were ground in extraction buffer (TRIzol^®^ reagent, Invitrogen) supplemented with β-mercaptoethanol (SIGMA-ALDRICH) and RNAse inhibitors (Ambion, Invitrogen) in 2 mL RNase free Eppendorfs (Qiagen). Total RNA was extracted using PureLink™ Micro-to-Midi Total RNA purification kit (Life Technologies). Residual genomic DNA was eliminated using DNAse for more accurate measurement of total RNA and more efficient cDNA synthesis. The DNAse (Promega) was inactivated properly prior to retro-transcription (RT) through phenol:chloroform:isoamyl purification. Total RNA was re-suspended in ultrapure water and measured with a NanoDrop 2000c spectrophotometer (Thermo Scientific, DE) and quality was checked using the Experion automated electrophoresis system (Biorad, CA, USA). An amount of 15 μg total RNA was used per RT reactions and fluorophore tagging was completed prior to the hybridisation on the microarray slide (Genisphere Array 350 3DNA™ and SuperScript II retro-transcriptase Invitrogen’ kit).

### cDNA microarray description, hybridisation and analysis

The cDNA microarray used in this study was developed by Kalujnaia *et al*. [23] for European eel (*Anguilla anguilla*) and was successfully used in a previous study on *A. rostrata*[24]. This array comprises 6144 expressed sequenced tags (ESTs) spotted in triplicate on glass silicate matrix lamella (GAPS II: Corning Inc, NY) by the service for microarray printing of the Biological Sciences School at Liverpool University. Initial ESTs were extracted from various tissues including brain, gills, guts and kidney.

### Experimental design, data acquisition and analyses

The experimental design consisted in paired comparisons where two individuals were hybridized simultaneously on each array using two fluorophores (cyanine3 and cyanine5). From the initial 12 individuals randomly sampled per group and later hybridized, 10 were retained for the analysis (total of 60 individuals) for standardization in terms of both numbers of samples and the quality of the hybridization signal. Glass eels correspond to samples analyzed at T0 and 10 samples from each location were compared for the final analysis, half with each different fluorophore (Cy3, Cy5). For the analysis after three months of common rearing (T3), each sample (now named elvers) was used for two comparisons in order to assess the relative role of origin, salinity conditions, and their interactions. Ten samples (five for each fluorophore) for each salinity condition and for each origin were compared.

Hybridized microarrays were scanned using the ScanArray Express equipment (Packard Biosciences). Images were scanned at a 10 μm resolution and saved as *TIF files. Signal intensity (532 nm for Cy3 and 635 nm for Cy5) was extracted using QuantArray^®^ (Perkin Elmer Life Sciences). Outlier spots and background noise were subtracted using the «Row Average Imputer» function and missing data were calculated through the «K-nearest neighbours» algorithm, both included in the R SAM software [25]. Once the background noise was subtracted, the average intensity of each transcript (gene, cDNA, in triplicate) was divided by the average intensity of its correspondent fluorophore (channel). A given gene was retained for further analyses if its intensity exceeded twice the average intensity plus twice the standard deviation of the negative control (empty spots) found on the same array with the same fluorophore. Base-2 logarithm was used to normalise the distribution of the average values retained, which were then normalized with the regional lowess method of the R/MANOVA package.

To detect gene expression differences among the tested groups, data were analysed using a mixed model ANOVA [26] and the R/MAANOVA package [27]. We tested for origin effects with the following model at T0 (Yijkl = μ + Ai + Dj + Ok + ϵijkl) and the following model for the experiments at T3 (Yijkl = μ + Ai + Dj + Ok + Sl + ϵijkl) where A: array; D: Dye; O: Origin; S: Salinity. The "Array" and "Sample" terms are treated as random whereas "Dye" and "Origin" are fixed terms. A permutation-based F-test (Fs statistic, with 1000 sample ID permutations) was performed and maximum likelihood was used to solve the mixed model equations [28]. We tested for origin type effects with the ANOVA model and used the p-values to determine the significance of differential expression. In order to limit type I error, a False Discovery Rate correction (FDR = 0.05 and 0.10) was applied within the R/MAANOVA package to account for multiple testing. F tests with 1000 permutation and maximum-likelihood restriction were used to solve the mixed model.

### Gene clustering

In order to reduce type II errors, a hierarchical clustering analysis using the complete-linkage method was performed on the entire raw fold-changes dataset. This method creates compact classes of very similar observations in terms of co-variation in expression. The number of classes to be considered was established using the cubic clustering criterion [29] implemented in SAS 9.2. This revealed an optimized grouping into seven gene clusters (see Results). Then, the GLM procedure in SAS was applied to test for overall differences among clusters followed by a K-means analysis to identify which clusters differed among themselves (XLSTAT, 1995). The reaction norms for the different clusters were based on gene expression mean fold change difference between any two groups compared. Here, the group which was the least expressed relative to the other was set on the zero base line, for the purpose of the comparison in relative terms.

### Annotation

Transcript sequences were annotated using the software «*Blast2GO PRO*» [25]. Sequences were uploaded in Blast2GO and analyses were run as recommended by the developer. First, the loaded sequences were run through *blastx program* on non-redundant database (nr Blast DB from QBlast-NCBI), 1.0E-3 Blast ExpectValue, with the maximum of 20 Blast Hits, word size of 3, low complexity filter, HSP length cutoff of 33. Secondly, the GO-Mapping sequences step was performed to link BLAST Hits to the functional information stored in the Gene Ontology database. Blast2GO uses different public resources provided by the NCBI, PIR, and GO to link the different protein IDs to the information stored in the GO database. All annotations are associated to Evidence Code which provides information about quality of this functional assignment (Blast2GO support information). The third step consisted in the Annotation default parameters which were set as followed: 1.0 E-6 E-Value-Hit-Filter, 55 of Annotation CutOff, five of GO Weight and no Hsp-Hit Coverage CutOff. Annotation Expander (ANNEX) was run to use an additional Gene Ontology structure which suggested new biological processes and cellular component annotations, based on the genes’ existing ‘’Molecular-Function” annotations (Ref.: ANNEX by GOAT, the Gene Ontology Annotation Toolbox, http://goat.man.ac.uk/ Simen Myhre and Henrik Tveit). The fourth step was the Enzyme Code Mapping and Kegg Pathway information, followed by InterProScan. Once all annotation steps were completed, GO and InterProScan informations were merged before running GO-slim.

## Results

### Gene annotations and clustering

Genes that significantly differed in expression in the various comparisons (see below) were identified through homology in translated proteins in non-redundant NCBI, non-redundant (nr) and GO mapping. Since these putative proteins have been mainly characterized in a few model organisms, and only represents partial information in cDNA microarrays, we considered them as part of “gene families” rather than precise unigenes and avoided over-interpretation about their biological function as this must await further functional characterization in terms of ecological annotation [30]. The proportion of annotated gene families varied between 28% and 68% (mean = 47%) depending on comparisons (see Results below). As hypothesized by Kalujnaia *et al*. [23], the high number of unidentified sequences obtained probably reflects the large number of 3’-untranslated region fragments that were spotted on the microarray. These gene families were then classified into eight functional categories previously described in Kalujnaia *et al*. [23] in their transcriptomic study of osmoregulation and development regulation in European eel (*A. anguilla*). These functional categories are: Cell protection/immunity, Detoxification, Energy metabolism/respiration, Growth/differentiation/development, Membrane transporters/carrier proteins, Signal transduction, Structural/junctional complex, and Transcription/translation. Table 1 presents the list of differentiated gene families within each of these functional categories, as well as their representation across different comparisons based on single transcript analysis and in each cluster based for the hierarchical clustering analysis (details below). Gene families that have previously been reported in other relevant studies on the transcriptomics of osmoregulation in fish are also indicated, with an emphasis on the study of Kalujnaia *et al*. [23] on European eel.

**Table 1:** List of differentiated genes within general function categories and their representation across different comparisons based on single gene analysis and in each cluster based for the hierarchical clustering analysis

**General function categories**	**Comparisons**					**Cluster**						
Gene name (within families)												
**Cell protection and immunity**	**T0**	**BW**	**FW**	**MR**	**GRB**	**1**	**2**	**3**	**4**	**5**	**6**	**7**
Alpha2-macroglobulin	1	~	1	1	~	_	_	_	_	_	_	_
*Aminopeptidase	5	1	12	15	6	4	1	_	7	1	_	_
Bactericidal/permeability-increasing protein	~	~	~	~	~	_	_	1	_	_	_	_
Complement component	2	~	1	~	1	_	_	1	_	_	_	_
Dipeptidyl peptidase 4	1	1	~	~	~	_	_	_	_	_	_	_
Elastase inhibitor	~	~	~	~	~	1	_	_	_	_	_	_
Heat shock protein	2	~	~	1	~	_	_	_	_	_	_	_
Hemolytic toxin avt-1	~	~	~	~	~	1	_	3	_	_	_	_
*Lactose-binding lectin I-2	~	~	~	~	~	_	_	2	_	_	_	_
Laminin receptor	1	~	~	~	1	_	_	_	_	_	_	_
*Lectin (β-galactoside-binding)	1	~	~	3	2	1	1	_	_	_	_	_
Lysozyme G	1	~	~	~	1	_	_	_	_	_	_	_
*Beta-2 Microglobulin	~	~	~	~	~	_	1	_	_	_	_	_
*Mucin	1	~	1	1	1	2	_	1	_	1	_	_
^£^*Myosin regulatory light chain kinase	1	1	~	~	~	_	_	_	_	_	_	_
Nattectin	~	~	~	~	~	_	_	_	_	11	_	_
*Pentraxin	~	1	1	1	~	_	1	_	_	_	_	_
Proteasome (link with ubiquitin)	1	~	~	1	1	_	_	1	_	_	_	_
*Serine (or cysteine) proteinase inhibitor, clade B	1	2	~	~	~	_	_	_	_	_	_	_
Superoxide dismutase	~	~	~	~	~	_	_	1	_	_	_	_
T-cell receptor beta chain	~	~	~	~	~	1	_	_	_	_	_	_
*Tumor necrosis α factor	~	~	~	~	~	1	_	_	_	_	_	_
**Detoxification**	**T0**	**BW**	**FW**	**MR**	**GRB**	**1**	**2**	**3**	**4**	**5**	**6**	**7**
Amine oxidase	~	~	2	~	1	_	_	_	_	_	_	_
*Glutathione S-transferase	1	1	~	~	1	_	_	1	_	_	_	_
*Metallothionein 1	~	~	1	1	~	_	_	_	_	_	_	_
**Energy metabolism & respiration (digestion)**	**T0**	**BW**	**FW**	**MR**	**GRB**	**1**	**2**	**3**	**4**	**5**	**6**	**7**
*Adenine nucleotide translocator	2	~	~	~	~	_	_	_	_	_	_	_
Adenylate cyclase	1	~	~	~	~	_	_	_	_	_	_	_
Ataxin	1	~	~	~	~	_	_	_	_	_	_	_
ATP synthase	1	~	~	1	1	1	_	_	_	_	_	_
ATP h + transporting f1	~	~	~	~	~	_	_	1	_	_	_	_
Chitinase	~	~	~	1	1	_	1	_	_	_	_	_
Cystatin	1	~	1	1	~	_	_	1	_	_	_	_
*Cytochrome c oxydase	22	10	7	3	17	10	_	6	_	_	_	_
Enolase	1	1	2	4	~	_	_	_	3	_	_	1
Ferritin, heavy polypeptide	3	~	~	1	2	_	_	_	_	_	_	_
Gastrotropin	1	2	1	~	2	_	_	_	_	_	_	_
*GDP-fucose transporter	~	~	~	~	~	1	_	_	_	_	_	_
Glycosyltransferase	~	~	1	1	1	_	_	_	_	_	_	_
Glutaminyl-tRNA synthase	1	~	1	~	~	_	_	_	_	_	_	_
*Hemoglobin cathodic alpha chain	2	~	2	~	~	_	_	_	_	_	_	_
Katanin	2	~	~	1	~	_	_	_	_	_	_	_
Lactate dehydrogenase	~	1	~	~	~	_	_	_	_	_	_	_
Lipase	2	1	3	~	1	1	1	_	_	_	_	_
*Maltase-glucoamylase	3	1	1	3	2	_	1	3	_	_	_	_
^¥^*Na.K-ATPase	1	2	~	1	1	_	_	_	_	_	_	_
*NADH dehydrogenase	1	2	~	~	1	1	_	_	_	_	_	_
Pyridine nucleotide-disulphide oxidoreductase	1	~	~	~	~	_	_	_	_	_	_	_
RAG1-activating protein 1 homolog	~	~	1	~	~	_	_	_	_	_	_	_
SH3 domain binding glutamic acid-rich-like protein	1	~	~	1	1	_	_	1	_	_	_	_
*Solute carrier 15	2	~	2	~	5	_	_	1	_	_	_	_
Triosephosphate isomerase	~	~	1	1	~	_	_	_	_	_	_	_
UGT1ab	~	~	~	~	~	1	_	_	_	_	_	_
**Growth, differentiation, development**	**T0**	**BW**	**FW**	**MR**	**GRB**	**1**	**2**	**3**	**4**	**5**	**6**	**7**
Actinin	1	~	~	~	~	_	_	_	_	_	_	_
Basic helix-loop-helix protein 2 (bHLHB2) Class B	~	~	~	1	~	_	_	_	_	_	_	_
Calpain	1	~	~	~	1	_	1	_	_	_	_	_
Caspase	1	~	~	~	~	_	_	_	_	_	_	_
*Cathepsin	2	~	~	2	1	_	_	2	_	_	_	_
CCAAT/enhancer binding	~	~	1	~	~	1	_	_	_	_	_	_
Chemotaxin (leucocyte cell derived)	2	16	2	~	22	6	20	_	_	_	_	_
^¥^*Claudin	5	1	3	3	6	3	_	3	_	_	_	_
*CUB and zona pellucida-like domain-containing protein 1	~	~	2	~	2	_	_	_	_	_	_	_
*Elongation factor 1-alpha	2	~	3	~	1	_	1	_	_	_	_	_
Ependymin precursor	~	~	~	~	~	1	_	_	_	_	_	_
Epithelial membrane protein	~	~	~	~	~	_	_	1	_	_	_	_
Galectin	3	~	2	5	~	_	_	_	_	_	_	_
GTPase slip-gc	~	~	~	~	~	1	_	_	_	_	_	_
Heart of glass protein	~	~	~	~	~	1	_	_	_	_	_	_
*Keratin	3	4	3	2	3	_	_	2	_	_	_	2
Neuronal guanine nucleotide exchange factor	~	~	1	~	~	_	_	_	_	_	_	_
*Nephrosin astacin-like metalloendopeptidase	1	~	~	~	~	_	_	_	_	_	_	_
*Profilin	1	2	~	1	1	_	3	1	_	_	_	_
^¥^S100 calcium binding protein	4	~	2	1	~	2	_	2	_	_	1	_
*Sciellin isoform a	~	~	~	~	1	1	_	_	_	_	_	_
Transcription factor INI	~	~	1	~	~	_	_	_	_	_	_	_
*Uroplakin 2	2	~	1	2	~	_	_	1	_	_	_	_
Zona pellucida glycoprotein	1	~	1	~	~	_	_	11	_	_	_	_
**Membrane transporters and carrier proteins**	**T0**	**BW**	**FW**	**MR**	**GRB**	**1**	**2**	**3**	**4**	**5**	**6**	**7**
Annexin	5	1	3	~	2	_	_	1	_	_	_	_
*Apolipoproteins	7	4	1	12	6	4	_	1	1	_	2	1
Cytolysin	~	~	~	~	~	_	_	4	_	_	_	_
Glutamyl aminopeptidase	~	~	~	~	~	1	_	_	_	_	_	_
**Signal transduction**	**T0**	**BW**	**FW**	**MR**	**GRB**	**1**	**2**	**3**	**4**	**5**	**6**	**7**
*Angiotensin	1	~	~	~	1	_	_	_	_	_	_	_
*Cytochrome P450	2	~	~	~	~	_	_	_	_	_	_	_
*Ictacalcin	~	~	~	~	~	_	_	4	_	_	_	_
^¥^*Inositol(myo)-1(or 4)-monophosphatase 1	~	1	~	1	1	1	_	_	_	_	_	_
*Interferon-induced gene 2	~	~	~	~	~	2	_	6	_	_	1	_
KDELR1	1	4	~	7	7	_	_	9	_	_	_	_
^£^Protein kinase C delta type	~	~	~	~	~	_	_	1	_	_	_	_
Myelin	~	~	~	~	~	_	_	2	_	_	_	_
*Prolactin	2	~	~	~	~	_	_	_	_	_	_	_
*Secretogranin III	1	~	~	~	~	_	_	_	_	_	_	_
Selenium binding protein 1	1	~	~	1	1	_	_	_	_	_	_	_
T-lymphocyte maturation-associated protein	2	~	~	~	2	_	_	_	_	_	_	_
Ubiquitin	1	~	~	~	~	_	_	_	_	_	_	_
*Zymogen granule	~	~	~	~	~	1	_	_	_	_	_	_
**Structural/junctional complex**	**T0**	**BW**	**FW**	**MR**	**GRB**	**1**	**2**	**3**	**4**	**5**	**6**	**7**
Actin alpha 1, skeletal muscle	1	~	~	~	~	_	_	_	_	_	_	_
Cofilin	2	~	1	2	~	2	_	3	_	_	_	_
*Collagen	1	~	2	~	1	1	_	_	_	_	_	_
High choriolytic enzyme 1 precursor	~	~	~	~	~	_	2	_	_	_	_	_
Mesothelin	1	~	~	~	~	1	_	_	_	_	_	_
Type i cytoskeletal protein	~	~	~	~	~	1	_	_	_	_	_	_
**Transcription/translation**	**T0**	**BW**	**FW**	**MR**	**GRB**	**1**	**2**	**3**	**4**	**5**	**6**	**7**
Neurogenic differentiation factor 1	~	~	~	~	1	_	_	_	_	_	_	_
Polymerase	1	~	~	~	~	_	_	_	_	_	_	_
PHD finger-like	~	~	~	~	~	1	_	_	_	_	_	_
RNA-binding protein	~	~	~	~	~	1	_	_	_	_	_	_
*Ribosomal protein	26	7	27	8	17	8	7	10	_	_	1	_
Translation initiation factor	1	~	~	~	~	1	_	_	_	_	_	_

*Indicates homologs identified in the study of Kalujnaia et al. [48], ^¥^indicate those identified in Whitehead et al. (2011) and ^£^those identified in Hoffman et al. [48].

The hierarchical clustering analysis, performed on all genes detected that were common to all eel groups compared, was resolved by seven distinct clusters of genes that co-varied in expression (Table 2). The total number of transcripts per cluster varied between 183 (cluster 1) and 8 (cluster 7) whereas the number of annotated transcripts varied between 93 (cluster 3) and 4 (cluster 7). Annotated transcripts were assigned to different gene families that represented between one to eight functional categories depending on clusters. Thus, most gene families were shared between two clusters or more but one private gene family (detoxification) was observed for cluster 3. Details on the gene families belonging to different functional categories and represented in each cluster are presented in Table 1. These seven clusters were used for subsequent analyses, as detailed below.

**Table 2:** Distribution of gene families comprising annotated genes across the seven clusters

**Cluster**	**1**	**2**	**3**	**4**	**5**	**6**	**7**
Number of genes included	183	113	157	20	19	16	8
Number of annotated genes	71	43	93	11	13	5	4
Cell protection/immunity	7	4	7	1	3	0	0
Detoxification	0	0	1	0	0	0	0
Energy metabolism/respiration	6	3	6	1	0	0	1
Growth/differentiation/development	8	4	8	0	0	1	1
Membrane transporters/carrier proteins	2	0	3	1	0	1	1
Signal transduction	3	0	5	0	0	1	0
Structural/junctional complex	4	1	1	0	0	0	0
Transcription/translation	4	1	1	0	0	1	0

### Differentiation between glass eels at time of capture

The number of uniquely expressed transcripts varied between 1400 and 2179 depending on comparisons (Table 3). The null hypothesis of no difference of gene expression between glass eels from the two origins at the time of capture in natural conditions was rejected whereby 883 unique transcripts showed significant difference in expression between individuals from GRB and MR. Of these, 57% were over-expressed in MR relative to GRB. The number of transcripts passing both FDR thresholds were comparable to that observed based on p-value. A total of 154 unique transcripts were annotated and these were assigned to different gene families which represented all eight functional categories (Tables 1 and 4). The three functional categories that were the most represented in terms of absolute number of gene families were: Cell protection/immunity, Energy metabolism/respiration, and Growth/Differentiation/Development.

**Table 3:** **Numbers of transcripts detected in all comparisons**, **numbers of differentially expressed transcripts and their proportions**

**Comparisons**	**All detected transcripts**	**All differentially expressed transcripts (% of all detected transcripts)**			**Overexpressed transcripts**	
**Between origins**		(p < 0.05)	(FDR 5%)	(FDR 10%)	MR	GRB
**T0**	2179	883 (41%)	898 (41%)	1144 (53%)	504 (57%)	379 (43%)
**BW**	1633	259 (16%)	18 (1%)	75 (5%)	116 (45%)	143 (55%)
**FW**	2094	422 (20%)	140 (7%)	249 (12%)	194 (46%)	228 (54%)
**Between environments**					BW	FW
**MR**	1400	453 (32%)	341 (24%)	469 (34%)	226 (50%)	227 (50%)
**GRB**	1794	501 (28%)	326 (18%)	581 (32%)	263 (53%)	238 (47%)

Legend: T0 is the comparison between glass eels captured at the river mouth prior to the beginning of the rearing experiment. GRB is for glass eels and elvers captured at the Grande-Rivière-Blanche river mouth and MR from Mira River. Glass eel were then reared in brackish water (BW) or fresh water (FW). All detected transcripts are the cDNA spots from the printed 6144 on the microarrays slide. Numbers of genes under the p-value are those for which their signal is significantly different between groups in the comparisons and the proportion over all detected transcripts (%). Numbers of genes under the FDR are the ones predicted to be false positives and the proportion over all detected transcripts (%). The numbers of genes in the ‘’Overexpressed transcripts” are those which were over-represented in a group compared to the other and their proportion (%) over the total (p < 0.05).

**Table 4:** Distribution of super gene families belonging to the different functional categories across the five comparisons

**Comparison**	**T0**	**BW**	**FW**	**MR**	**GRB**
Number of differentiated transcripts	883	259	422	453	501
Number of annotated transcripts	154	67	96	91	129
Cell protection/immunity	12	5	5	7	7
Detoxification	1	1	2	1	2
Energy metabolism/respiration	19	8	12	12	12
Growth/differentiation/development	14	4	12	8	9
Membrane transporters/carrier proteins	2	2	2	1	2
Signal transduction	8	2	0	3	5
Structural/junctional complex	4	0	2	1	1
Transcription/translation	3	1	1	1	2

**Figure 1:**
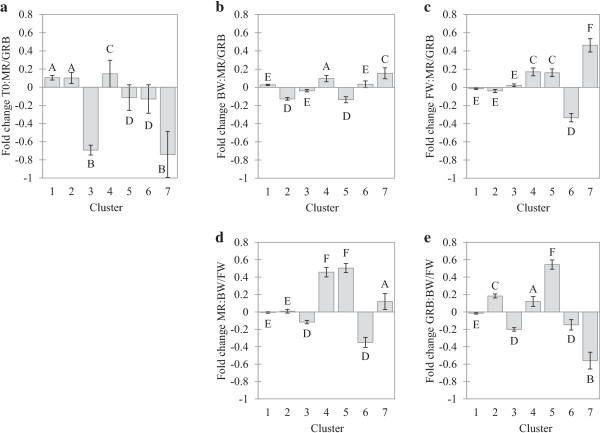
**Cluster mean fold change in Log-2 (and 95% confidence limits).** From left to right: **(a)** between glass eels at the river mouth (T0: MR/GRB), **(b)** between elvers of both origins after three months rearing in BW and **(c)** in FW, **(d)** between environments for elvers from MR, and **(e)** GRB. GLM procedure was done, followed by K-means analysis where clusters with the same letter are not significantly different (α = 0.05).

As for differentiation at individual transcripts, the null hypothesis of no difference in gene clusters that co-varied in expression was rejected. Figure 1a illustrates mean fold changes (in Log2) for transcripts included in each of the seven clusters and provides a general picture of how these differed between origins. The GLM procedure revealed a highly significant difference between mean expression level among the seven clusters (F = 123.69, F < 0.0001). K-means test indicated that the seven clusters formed three distinct groups (A:1,2; B:3,7: C4, D:5–6). The most salient result of this comparison was the pronounced down-regulation of clusters 3 and 7 in MR relative to GRB glass eels by approximately 60%. All eight functional categories are represented in cluster 3, among which three are shared with cluster 7 (Energy metabolism/respiration; Growth/differentiation/development; Membrane transporters/carrier proteins) (Table 2). Two annotated gene families were common to both clusters (Keratin and Apolipoproteins) (Table 1), and both were previously reported to show differences between European silver eels acclimated to different salinity conditions [23]. Other gene families belonging to cluster 3 that were both represented by several transcripts and that were also reported by Kalujnaia *et al*. [23] were Cytochrome c oxydase, Maltase-glucoamylase, Claudin, Cathepsin, Ictacalcin, and Ribosomal protein (Table 1).

### Differentiation between eels from distinct origins reared in fresh and brackish water

Significant differences between the two origins were maintained after three months of rearing in common environment but were less pronounced than at time of sampling in natural conditions. Thus, 259 and 422 unique transcripts showed differences in expression between origins when reared in BW and FW, respectively (Table 3). Of these, 55% were significantly over-expressed in GRB relative to MR in BW whereas this was the case for 54% in FW. However, the number of significant transcripts passing both FDR thresholds was substantially smaller, particularly in the BW comparison. Overall then, there was a trend towards less pronounced gene expression between origins in BW relative to freshwater. In the BW comparison, 67 of the differentiated transcripts were annotated and these represented seven of the eight functional categories (Table 4). In the FW comparison, 96 of the differentiated transcripts were annotated and these also represented seven of the eight functional categories. As in T0, the three functional categories that were the most represented in both BW and FW comparisons in terms of absolute number of gene families were: Cell protection/immunity, energy metabolism/respiration, and Growth/Differentiation/Development. The list of gene families that differed between origins in both environments and that were also reported by Kalujnaia *et al*. [23] was comparable to those observed at T0, comprising: Aminopeptidase, Cytochrome c oxydase, Maltase-glucoamylase, Claudin, Keratin, and Apolipoproteins, Ribosomal protein (Table 1).

We then tested for differentiation between origins depending on the rearing environment after three months (T3). Figure 1b illustrates mean fold change differences between MR and GRB for each of the seven clusters when reared in BW whereas Figure 1c represents the same comparison in FW. The GLM procedure revealed a highly significant difference between mean expression level among the seven clusters, both in BW and FW environment (respectively F = 88.73 and F = 85.16, p < 0.0001). The K-means test indicated that the behavior of some of the clusters varied between rearing conditions whereas others were similar. Thus, clusters 1 and 3 showed no clear difference in expression between origins both in BW and FW environments whereas clusters 4 and 7 were up-regulated in MR for both environments but the fold change for both clusters tended to be higher in FW than BW. Thus, there was a limited plastic response in expression for these genes. In contrary, the other three clusters showed a contrasted pattern of differentiation depending on the environment. For BW, cluster 2 was down-regulated in MR whereas no clear difference between origins was observed in FW. Conversely, cluster 6 was down-regulated for FW in MR whereas no clear difference in expression was observed for BW. Cluster 5 was down-regulated for BW and up-regulated in FW for MR. In addition to functional categories comprised in cluster 7 and defined above, genes that showed up-regulation for both environments in MR belonged to the Cell protection/immunity category (Table 2). This category was represented only by Aminopeptidase which was also reported by Kalujnaia *et al*. [23] (Table 1). Genes of cluster 5 presented the most pronounced pattern of plasticity, and all belonged to the Cell protection/immunity functional category represented by three gene families: two reported by Kalujnaia *et al*. [23] Aminopeptidase, Mucin, as well as Nattectin (Table 1). Genes belonging to cluster 6 were also plastic and represented four functional groups: Growth/differentiation/development, Membrane transporter/carrier proteins, Signal transduction and Transcription/translation (Table 2). These comprised four gene families that were all reported by Kalujnaia *et al*. [23]: S100 calcium binding protein, Apolipoproteins, Interferon-induced gene 2, and Ribosomal protein (Table 1).

Another way to look at environmental effect on the plasticity of gene expression is to compare the extent of differentiation between FW and BW within a same origin. Thus, 32% and 28% of detected transcripts showed differences in expression between salinity conditions for MR and GRB, respectively (Table 3). Of these, 50% were over-expressed in BW relative to FW for MR whereas this was the case for 53% for GRB. The number of significant transcripts passing both FDR thresholds was important although lower to those observed with p-values. Thus, at T3, there were more differences in expression between environments than between origins based on absolute number of differentially expressed transcripts. The number of differentiated transcripts between environments was comparable for both origins and these represented all eight functional categories (Table 4). There were numerous gene families that differed in expression between environments for both origins and that were also reported to show differences between European silver eels acclimated to different salinity conditions [23]. Those represented by the largest number of transcripts were: Aminopeptidase, Lectin (β-galactoside-binding), Cytochrome c oxydase, Maltase-glucoamylase, Claudin, Keratin, Apolipoproteins, and Ribosomal protein (Table 1). Na.K-ATPase (one transcript) also differed in expression for both origins. It is also noteworthy that seven transcripts of the KDELR1 (not reported in previous studies) gene family differed in expression for both origins. Finally, a few gene families showed contrasted patterns between origins. In particular, many more transcripts of Cytochrome c oxydase were differentially expressed between environments in GRB vs. MR (17 vs. 3). Also, five transcripts of Solute carrier 15 and 22 transcripts of Chemotaxin differed in expression in GRB vs. none in MR. Finally; there were twice as many transcripts of Apolipoproteins differentially expressed in MR vs. GRB (12 vs. 6).

**Figure 2:**
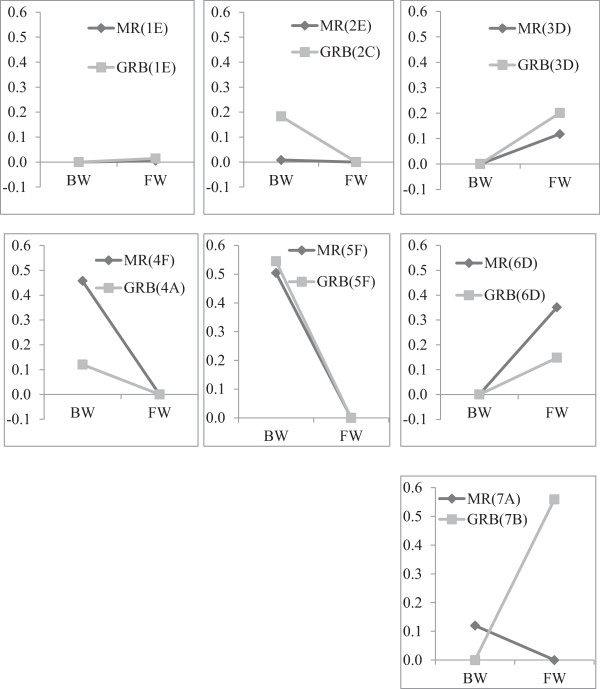
**Clusters genomic reaction norm**, **where MR and GRB absolute fold changes are illustrated on the same standardised scale.** K-mean categories defined in Figure 1 are given for each cluster.

We then compared the plastic response of the seven gene clusters between environments for eels from the two origins. Figure 1d illustrates mean fold change differences between environments for MR and Figure 1e represents the same comparison for GRB. The GLM procedure revealed a highly significant difference between mean expression level among the seven clusters, both for MR (F = 155.10, F < 0.0001) and GRB (F = 196.70, F < 0.0001). Again, the K-means test indicated that the plastic response to different rearing conditions varied among clusters, with four different cluster groups observed in MR and all seven gene clusters showing a different plastic response in GRB. Variation between rearing environments for clusters 1 to 6 were marginally correlated between MR and GRB (r^2^ = 0.646, F = 7.293 and p = 0.054), suggesting similar directionality in plastic response between origins to environmental conditions for these genes. In contrast, cluster 7 showed a pronounced gene-by-environment interaction whereby genes of this cluster were highly overexpressed in FW relative to BW water for GRB (fold change = 1.49) whereas they slightly tended to be overexpressed in brackish water in MR (mean fold change = 1.07). These differences translated into contrasted genomic reaction norms between origins for cluster 7 (Figure 2). Variable patterns of genomic reaction norms between origins were observed among the other six clusters. Cluster 1 showed no plastic response for both origins, cluster 2 showed no plastic response in MR but was overexpressed in BW relative to FW in GRB, translating origin-environment interaction. Clusters 3 and 6 were overexpressed in FW for both origins, whereas clusters 4 and 5 also showed a pronounced plastic response, both being overexpressed in BW for both origins. For cluster 4, however, the plastic response was more pronounced in MR than GRB, thus also translating origin-environment interactions. Thus, gene families that showed the most pronounced differences in genomic reaction norms between origins belonged to clusters 2, 4 and 7, the most important being: Lectin, Pentraxin, Maltase-glucoamylase, Elongation factor 1-alpha, Profilin, Chemotaxin, Aminopeptidase, Enolase, Keratin, Apolipoproteins and Ribosomal protein, all reported by Kalujnaia *et al*. [23] except for Chemotaxin (Table 1).

## Discussion

Since panmixia precludes any possibility for long-term local adaptations, a next step towards understanding how American eel can cope with highly heterogeneous environments is to identify factors explaining the pronounced local variations in life history as well as physiological and ecological traits observed in this species throughout its range of distribution [31]. In order to address this issue from a gene expression perspective, we assessed the relative role of geographic origin, environment (salinity) and their interaction on transcription profiles observed both in natural and controlled conditions. Our results revealed that all three factors play a role in explaining variation of gene expression in American eel.

### Transcriptomic divergence in natural conditions

Our results rejected the null hypothesis of no differences in gene expression between glass eels collected at same sizes and developmental stage, at the same time of arrival at different river mouths just prior to entering freshwater, thus avoiding differential effect of time spent in freshwater before experiments [6]. Nearly 40% of the 2179 unique transcripts that were detected showed significant difference in expression between individuals from GRB and MR. We cannot rule out the possibility that transcriptomic differences between sites are due to differences in local environmental conditions at time of sampling, namely temperature. Thus, mean sea surface temperatures are higher along the coast of Nova Scotia than in the St. Lawrence estuary (http://www.class.ncdc.noaa.gov data base SST14NA). However, gene expression generally increases with temperature in fishes [32,33] whereas we observed a trend towards overexpression in GRB relative to MR. Also, both samples were taken in similar brackish water just at the mouth of both rivers. Thus, different temperatures and salinities encountered by glass eels at time of capture may not be the most parsimonious explanation for the observation of differential expression between origins. Alternatively, differential selective pressures associated with variable environmental conditions being encountered during marine dispersal can result in genetic differences between glass eels from different locations through the process of spatially varying selection as demonstrated by Gagnaire *et al*. (2012) [12]. Notwithstanding a role for environmental plastic response in explaining differential expression between glass eels of different geographic origins (see below), this first result along with evidence for local genetic differences at coding genes points towards a role for some genetic differences between glass eels from different origins. Admittedly, epigenetic effects could also be invoked in explaining the observed differential pattern of expression between origins and cannot be refuted [34], although there is at present very little empirical evidence for this in nature [35]. Also, given the impossibility to date to routinely perform artificial reproduction and rear American eel larvae beyond the age of about 10 days [36], it was not possible to perform crosses and rear F2 progeny to rigorously rule out possible environmental and maternal carry-over effects that could impact on the observed patterns of gene expression.

### Transcriptomic divergence in controlled conditions: comparisons with other studies

The experiment conducted over a three-month period revealed that transcriptomic differences were maintained in controlled conditions. Further evidence for differential response between young eels from GRB and MR was provided by the origin-environment interactions observed for some of the gene clusters. Overall, general patterns of gene expression between distinct origins in controlled conditions corroborate those we observed previously for growth between glass eels from these same two locations [6]. Moreover, growth differences between origins associated with sex-specific effects were also maintained after three years of common rearing and were similar to those observed in natural conditions (Côté et al. in revision).

The scale of differences observed here between eels of both origins is comparable and even more pronounced to what has been reported in other fish studies comparing the transcriptome of genetically distinct populations within a same species. In a comparison between genetically distinct resident and anadromous brook charr (*Salvelinus fontinalis*) reared in common conditions, Boulet *et al*. (2012) [37] observed that gene expression in the gills differed for 4% of the genes, a value comparable to what we observed after 3 months of common rearing. In a study of divergence in gene regulation at young life history stages of whitefish (*Coregonus* sp.), Nolte et al. (2009) [38] found that 8.6% of genes differed in expressions between dwarf and normal whitefish populations. In a study comparing the ecological transcriptomics of lake-type and riverine sockeye salmon (*Oncorhynchus nerka*), Pavey *et al*. (2011) [30] found that about 1% transcripts differed between these locally adapted populations to distinct habitat types. In a comparative analysis of gene transcription between genetically distinct populations of the Atlantic killifish (*Fundulus heteroclitus*), Whitehead et al. (2011) [39] found that 8.2% of the 6800 genes analyzed differed in expression between populations.

In summary, results to date suggest that local genetic differences between glass eels of different origins are at least partly due to spatially varying selection translating into transcriptomic differences (including different genomic norms of reaction), which could partly explain the phenotypic variance observed between eels from different habitats. This offers a plausible working hypothesis towards solving the apparent paradox between observations of important regional variation in life-history traits, demography, and ecology on the one hand and on the other hand, evidence for the existence of a single, randomly mating population in Atlantic eel.

### Functional interpretations

Since growth is a predominant phenotypic trait that differs between eels from GRB and MR, one should expect that genes potentially involved in growth functions differ between them as well. Indeed, this was the case for some genes involved. Thus, some gene clusters (clusters 4, 7) showed either a net difference between origins when reared either in FW or BW or showed an origin effect on plastic response to environmental conditions (clusters 5, 6). Some of the genes super-families found in these clusters could potentially influence growth, for instance Cytochrome c oxidase (COX) which is involved in the respiratory chain [40], Keratin which is involved in critical cells functions such as migration, size, growth, transport and proliferation [41], and Profilin-2 which enhances actin growth [42]. However, growth is a complex phenotypic trait with a highly polygenic basis such that it would be risky at best to attempt any further causal links between specific functions and variation in growth. Besides these super-families that could play a role in growth, many other functions differed in expression between eels from different origins, as reported also in other fish studies comparing genetically distinct locally adapted populations [30,37-39].

Many of the gene families that showed significant differences in expression or expressed different genomic norms of reactions between geographic origins overlapped with genes that showed significant differences between adult silver European eels from a same location acclimated to different salinity conditions [23]. Many of those genes do not have any obvious direct link to osmoregulatory functions, indicating that salinity conditions may impact on the expression of many biological functions, including cell protection, immunity, detoxification, energy metabolism, growth, differentiation and development, membrane transporters, signal transduction, structural and junctional complex as well as transcription and translation. Here, while we observed a plastic response to salinity conditions for many of the gene families represented (see discussion below), we also found that some of those genes showed a non-plastic response that differed between origins or differential plastic responses between origins.

### Genomic reaction norms and possible evidence for adaptive plasticity

These results rule out the general belief that all variation in phenotypes and habitat use observed in eel is purely plastic [43,44]. On the other hand, they also support an important role of phenotypic plasticity in explaining eel evolutionary success in coping with a very broad array of ecological conditions. [39]. Thus, we observed more differences in expression between environments within origin than between origins for both rearing environments. However, the extent of plasticity to different salinity conditions was highly variable among genes. First, it is noteworthy that the overall pattern of plasticity in expression was relatively similar between origins whereby variation between clusters 1 to 6 was marginally correlated (Figure 1d and Figure 1e). However, different clusters showed different patterns of plasticity. The 183 genes belonging to cluster 1 did not show any variation in any of the comparison and as such, represent the group of genes that were the least plastic in expression. Clusters 3, 5 and 6 showed similar plastic responses in both origins. Finally, clusters 2, 4 and 7 revealed distinct reaction norms between GRB and MR. Differential plastic responses to environmental conditions among genes have commonly been reported and used to identify genes that may be the most important in coping with different environments [23,32,33,45-47]. These studies typically identified a broad array of genes that vary in expression despite no *a priori* expectation based on functions. This was also the case here whereby we observed a plastic response in expression to salinity condition for genes belonging to many gene super-families and functional groups without any known *a priori* role in osmoregulation. Nevertheless, several individual genes potentially involved in osmoregulation were also identified (Table 3), such has prolactin, Na-K ATPase, and kinase C [23,39,48-50].

The different genomic reactions observed between eels from different origins could reflect an adaptive plastic response to the environment. This could be the case if they were causally linked with differential plastic responses in growth to these same salinity conditions we observed previously [6]. This, however, needs to be rigorously investigated in future studies. Determining how plastic developmental changes that occur in response to environmental conditions (e.g. growth) are coordinated at the molecular (e.g. transcriptome level) represents a major challenge to be addressed in future studies by combining ecology with developmental biology [51].

## Conclusions and perspectives

In summary, our results revealed that geographic origin, environment and the interaction between them play a role in explaining variation of gene expression in American eel. This indicates that phenotypic variation observed in American eel is not purely plastic but also involves genetically (and/or epigenetic) based differences between locations. Whether these differences are adaptive remains to be demonstrated. Yet, the extent and nature of differential gene expression between eels of different geographic origins compares to differences reported between genetically distinct and locally adapted populations in other species. Given the increasing availability of genomic resources for studying *Anguilla s*pecies, including genome sequences [52,53] large annotated EST libraries [54] and dense microarrays [55], it will be feasible to investigate into more details how plastic developmental changes that occur in response to environmental conditions are controlled at the molecular level, including the detection of epigenetic regulation mechanism [56], in link with genome-wide spatially varying selection caused by natural environmental heterogeneity [12], habitat degradation [22,57], selective exploitation [58] or predation [59], as well investigating the molecular basis of behavioral traits that may be involved in habitat selection [60]. Such knowledge is essential towards improving conservation strategies for this threatened species in Canada [61].

## Data availability

The data set supporting the results of this article is available from the Dryad Digital Repository: http://doi.org/10.5061/dryad.nq38s.

## Abbreviations

MR: Mira river; GRB: Grande Rivière Blanche; T0: Time of collection on the field; T3: Time of collection after three months of common rearing; LARSA: Laboratoire de Recherche en Science Aquatique; FW: Fresh water 2–3 ppt; BW: Brackish water 20–22 ppt; LT: Total body length; W: Wet mass; RT: Retro-transcription; ESTs: Expressed sequenced tags; Cy3 and Cy5: Cyanine 3 and cyanine 5 fluorophores; FDR: False discovery rate correction; Nr: Non-redundant; COX: Cytochrome c oxidase.

## Competing interests

The data of this paper are original and no part of this manuscript has been published or submitted for publication elsewhere. The authors have no competing interests in this study.

## Authors’ contributions

CLC conceived and coordinated of the study, captured, reared and processed the fish specimens used in this study, carried out the molecular study, statistics and draft the manuscript. SKM and GC designed, annotated, and provided the microarray. MC and LB participated in all the steps of the conception the study, coordination and helped to draft the manuscript. All authors read and approved the final manuscript.

## Acknowledgement

We are grateful to Guy Verreault (MRN) for his comments and field tips, to Eric Normandeau for laboratory and statistical assistance, Lucie Papillon, Laure Devine, Nathalie Brodeur and Hacène Tamdrari for field assistance, to Yvonne Carey from Atlantic Elver Fishery for gracefully providing dip nets, and to Mike Campbell from South Shore Trading Co. for providing samples and advice on elver rearing. The assistance of Serge Higgins and Jean-Christophe Therrien from LARSA was invaluable throughout the rearing experiment. We are also grateful to Laura Castonguay for proof-reading and improving the quality of language. This research was funded through the Department of Fisheries and Oceans Canada as well as a Discovery grant from the Natural Sciences and Engineering Research Council of Canada to LB. This is a contribution to the research program of Québec-Océan.
